# Available drugs and supplements for rapid deployment for treatment of COVID-19

**DOI:** 10.1093/jmcb/mjab002

**Published:** 2021-01-25

**Authors:** Danielle Cicka, Vikas P Sukhatme

**Affiliations:** 1 Department of Pharmacology and Chemical Biology, Emory University School of Medicine, Atlanta, GA, USA; 2 Department of Medicine, Department of Hematology and Medical Oncology, Morningside Center for Innovative and Affordable Medicine, Emory University School of Medicine, Atlanta, GA, USA

Effective treatment for COVID-19 remains elusive, though urgently needed in the current pandemic. Repurposing marketed therapies may be an effective strategy for finding treatments quickly and recently, *in vitro* and clinical testing of such therapies against severe acute respiratory syndrome coronavirus 2 (SARS-CoV-2) has skyrocketed. However, not all marketed drugs showing *in vitro* efficacy could achieve therapeutic concentrations in humans and discernment of drugs that have favorable pharmacokinetic properties can save time and resources for future studies. Here, we compile marketed therapies, including supplements, having antiviral activity with *in vitro*, *in vivo*, and/or clinical data against α and β coronaviruses into tables, alongside their pharmacokinetic properties. We point to several drugs or supplements available for immediate repurposing because they have achievable blood concentrations in humans well above their inhibitory concentrations against coronaviruses. This compilation may contribute to the implementation of rapid future studies by narrowing the vast number of marketed drugs reported for potential efficacy against SARS-CoV-2 on the basis of their pharmacokinetic properties and published coronavirus data.

## Introduction

There is an urgent need for effective treatments for COVID-19, the clinical manifestation of SARS-CoV-2 infection. Over 83 million cases of COVID-19 and over 1.8 million deaths due to COVID-19 have been documented worldwide by early January 2021 (https://www.worldometers.info/coronavirus/). COVID-19 can spread through asymptomatic or symptomatic patients who may present with a wide variety of symptoms including dyspnea, cough, fatigue, and fever (Grant et al., 2020; Wiersinga et al., 2020). The fatality rate is difficult to calculate and depends on many factors including age, comorbid conditions, and gender, but is estimated to range from 0.03% to 30% and >30% mortality in the intensive care unit (Quah et al., 2020; Wiersinga et al., 2020). Current treatments include supportive care, dexamethasone, and remdesivir for hospitalized patients (Wiersinga et al., 2020). New treatments are urgently needed for both inpatient and outpatient cases. However, the development of novel pharmaceuticals from preclinical studies to Phase III clinical trials is often a long process, wrought with failures. Alternatively, marketed drugs may provide a favorable starting point for treatment discovery, since there are already indications of their safety, toxicity, and pharmacokinetic profiles in humans. Maintaining high ethical standards and stringent criteria for evaluating efficacy of repurposing drugs is crucial, which, when done correctly, may expedite the therapeutic discovery process in a crisis, saving time, money, and lives.

To harness the valuable information that researchers have produced in the face of previous outbreaks of coronaviruses and in the current pandemic, we have compiled the scientific literature on marketed therapies for the treatment of coronaviruses to prioritize therapies for further testing. This includes *in vitro* half maximal inhibitory concentrations (IC50s), animal studies, human studies, potential mechanisms of action, pharmacokinetic data, and *in silico* data, if available. Our analysis is different from previous works such as Arshad et al. (2020), as it focuses on marketed therapeutics and supplements with reported blood concentrations at least five times (5×) higher than their IC50s in coronavirus system and compiles clinical, *in vivo*, *in vitro*, and *in silico* data.

## Materials and methods

Coronaviruses are classified into four genera: α, β, γ, and δ. All seven coronaviruses known to cause respiratory infections in humans fall into the α or β genera, with SARS-CoV-2, SARS-CoV-1, and Middle East respiratory syndrome coronavirus (MERS-CoV) as members of the β coronavirus genera (Ye et al., 2020). Given sequence and biological similarities between the α and β coronaviruses, identifying and prioritizing marketed drugs that have *in vitro* or *in vivo* activity against these coronaviruses is a reasonable approach, while noting some distinct similarities between SARS-CoV-2 and SARS-CoV-1 (Jaimes et al., 2020; Ye et al., 2020). Therefore, to obtain our initial list of therapies, we conducted PubMed and Google Scholar searches using keywords such as ‘coronavirus’, ‘SARS2’, ‘COVID-19’, ‘SARS-CoV-2’, ‘repurposed drugs’, ‘treatment’, and ‘therapy’.

We compiled a list of available small molecules that have been tested *in vitro* against coronaviruses into tables. We further included an achievable maximal concentration (Cmax) in blood or plasma regardless if steady state is reached or lowest concentration before next dose (Ctrough) obtained in clinical studies of healthy human subjects or in studies of the dosing and treatment regimen in humans as generally indicated for the drug. In order to enhance chances of efficacy in humans, drugs with reported blood concentrations at least 5× greater than IC50 values obtained in cell culture against coronaviruses were further selected. If a drug is rapidly metabolized and the blood concentration of the metabolite was available, this was used for comparison to the IC50s and indicated in column 2 of Supplementary Table S1. Recognizing the variation between experimental IC50s, if one experimental IC50 fitted this criterion, the drug was considered a promising candidate. If SARS-CoV-2 data were not available, IC50s against other coronaviruses were used for selection. We compiled the studies testing efficacy of these candidates against coronaviruses including clinical data, *in vivo* studies, *in vitro* studies, *in silico* studies, and potential mechanisms of action.

We have assembled the information on each drug into three tables: Table 1 and Supplementary Tables S1 and S2. Table 1 includes the highest level of available data of drugs and supplements against SARS-CoV-2 or COVID-19. We chose the highest level of evidence to be randomized, double-blinded, controlled trials of COVID-19 patients, followed by other types of clinical trials for COVID-19 patients, *in vivo* SARS-CoV-2 studies, *in vitro* studies against SARS-CoV-2, and then clinical trials and *in vitro* studies on other coronaviruses. If the highest level of evidence for a drug or supplement did not show any efficacy, the therapy was not included. Supplementary Table S1 includes extended data of the drugs and supplements in Table 1 against any α or β coronavirus, including SARS-CoV-2, along with the reported blood concentrations used for the selection of these drugs and supplements. Supplementary Table S2 includes drugs and supplements without available data against SARS-CoV-2 or COVID-19 but showing achievable blood concentrations at least 5× higher than their IC50s against other coronaviruses. The drugs in these tables may be the most promising candidates for further *in vitro*, *in vivo*, or clinical evaluation of their activity against coronaviruses. They are arranged in alphabetical order in each table. Values were estimated if necessary and indicated by ‘∼’ prior to the value. Combination therapies are in orange text.

## Results

From our compilation of the literature on marketed drugs and supplements with efficacy against coronaviruses, several candidates stand out as fitting our criteria for favorable pharmacokinetic properties. These candidates with reported activity against SARS-CoV-2 are listed alphabetically in Table 1. However, as shown in Supplementary Table S2, oxaprozin and telavancin showed activity against other coronaviruses with blood concentrations 5× greater than their IC50s, but no peer-reviewed data against SARS-CoV-2 have yet been reported to our knowledge.

**Table 1:** The highest level of data available for drugs or supplements acting on the viral life cycle with blood concentrations greater than 5× their IC50s against SARS-CoV-2.

Drug or supplement name	Human data in coronavirus patients	Animal data	Cell culture data (IC50 in μM)
Baicalin	NA	NA	SARS-CoV-2 3CLpro inhibition IC50 = 6.41 (Su et al., 2020)
Dalbavancin	NA	SARS-CoV-2 mouse model treated with dalbavancin (130 mg/kg intraperitoneal on Day 0) and SARS-CoV-2 rhesus macaque model treated with dalbavancin (60 mg/kg on Day 0 and 30 mg/kg on Day 4) showed lower viral load and reduced histopathological injury compared to control (Wang et al., 2020a)	See Supplementary Table S1
Dipyridamole	Prospective, open-label, randomized, controlled study of COVID-19 patients showed those receiving dipyridamole treatment (50 mg 3×/day) had higher clinical remission rates, decreased D-dimer, and increased lymphocytes and platelets compared to control patients (Liu et al., 2020)	NA	See Supplementary Table S1
Eltrombopag	NA	NA	SARS-CoV-2 IC50 = 8.27 (Vero cells) (Jeon et al., 2020) SARS-CoV-2 IC50 = 8.38 (Calu-3 cells) (Ko et al., 2021)
Favipiravir	Prospective, open-label, randomized, controlled study of COVID-19 patients receiving favipiravir (1800 mg 2×/day on Day 1, then 800 mg 2×/day) showed shorter time to clinical improvement compared to control (Udwadia et al., 2020) Prospective, open-label, randomized, controlled study of COVID-19 patients showed those receiving favipiravir (1600 or 2200 mg on Day 1, then 600 mg 3×/day) had no significant reduction in viral load compared to control (Lou et al., 2020)	See Supplementary Table S1	See Supplementary Table S1
Mycophenolate mofetil/myco- phenolic acid	NA	See Supplementary Table S1	Mycophenolate acid against SARS-CoV-2 IC50 = 0.87 (Vero/TMPRSS2 cells) (Kato et al., 2020) Mycophenolic acid against SARS-CoV-2 IC50 = 0.101 (Vero E6 cells) (Wan et al., 2020)
Nafamostat	Case reports and retrospective, uncontrolled study of COVID-19 patients treated with nafamostat (Doi et al., 2020a, b; Jang and Rhee, 2020; Osawa et al., 2020)	NA	See Supplementary Table S1
Nitazoxanide	See Supplementary Table S1	NA	SARS-CoV-2 IC50 = 2.12 (Vero E6 cells) (Wang et al., 2020b)
Remdesivir	Prospective, randomized, controlled trial of COVID-19 patients showed those receiving remdesivir (200 mg intravenous on Day 1, then 100 mg/day) had faster time to clinical improvement, but not statistically significantly different compared to control (Wang et al., 2020c) Prospective, randomized, controlled study of COVID-19 patients showed those treated with remdesivir (200 mg intravenous on Day 1, then 100 mg/day) had shorter time to recover compared to control (Beigel et al., 2020)	See Supplementary Table S1	See Supplementary Table S1
Sulfadoxine	NA	NA	SARS-CoV-2 IC50 = 35.37 (Vero E6 cells) (Touret et al., 2020)
Teicoplanin	Retrospective, uncontrolled study of hospitalized COVID-19 patients receiving teicoplanin (600 mg/day) (Ceccarelli et al., 2020)	NA	See Supplementary Table S1

NA, not available.

A brief commentary on the drugs is available in Supplementary Table S3 including their indications and common side effects that typically occur at an incidence of 1% or greater according to Drugs.com (https://www.drugs.com/), unless otherwise noted.

Based on the route of administration, toxicity profile, and therapeutic index, these drugs would be more or less appropriate in the outpatient or inpatient setting.

## Limitations

We focused on compiling a useful, but simple table on potential drugs for repurposing for COVID-19, utilizing IC50s and known achievable blood concentrations. However, this strategy has some limitations and we encourage reading of the primary literature for further information. In particular, these tables do not contain an exhaustive compilation of pharmacokinetic properties of these therapies for simplicity and a more complete evaluation of the pharmacokinetic properties of these drugs should be done prior to further studies. For example, the achievable concentration in the lung may be different from that in the peripheral blood, the reported concentration in the blood may not reflect the fraction of drug unbound in plasma, and the variable and transient nature of Cmax does not depict tissue exposure to a drug over time. Route of administration, drug‒drug interactions, clinical characteristics of patients, and dose scheduling may alter the achievable concentrations in the lung or peripheral blood as well. Some drugs, notably mycophenolate mofetil, nitazoxanide, and remdesivir, are quickly metabolized and studies did not always determine whether the parental compound or metabolite were responsible for antiviral activity *in vitro*, which complicates determining whether their IC50s are achievable in humans. Further, clinical trials involve the study of patients with a complex set of treatments, comorbidities, and outcomes, which are not captured in this table but could provide valuable insight into mechanisms of action or differences in effectiveness. These tables also do not contain information on ongoing clinical trials as the focus is on reported data for these therapies. Additionally, supplements are not FDA approved and have not necessarily been studied in clinical trials. These small molecules should be further evaluated for their safety profiles and pharmacokinetic parameters if needed.

Also, IC50 does not give a complete picture of the effectiveness of a drug against coronavirus. Though we chose drugs with IC50s at least 5× lower than their known achievable blood concentrations, the dose‒response curve and IC90 for each drug may paint a more clinically relevant picture for dosage. Further, IC50s against coronaviruses differ based on assay. There are many variations of *in vitro* assays to determine a small molecule’s efficacy against viruses utilizing different readouts, conditions, and various viral strains. Some drugs may have effects both on the viral life cycle and on the immune response and therefore may have different effects *in vivo*. Many of the IC50s listed are obtained through experiments that do not utilize primary human lung cells and it has been noted in previous studies that the IC50 may vary markedly based on cell lines used (Hoffmann et al., 2020). Animal and human studies are needed to determine the true efficacy of these drugs against SARS-CoV-2.

## Speculations

We speculate that some of these small molecules may be candidates for further research as therapies for COVID-19, since their IC50s are well below their achievable blood concentrations. Combining drugs with differing mechanisms may also show synergistic effects and reduce emergence of resistance. Further, COVID-19 is a complex disease with a strong immune component (Blanco-Melo et al., 2020; Qin et al., 2020). Therefore, we speculate that a combination of host-directed and viral-directed therapies with achievable inhibitory concentrations in humans may be particularly effective treatment for COVID-19. Timing of these therapeutic combinations may be important as the immune response may vary throughout the clinical disease course (Shi et al., 2020). Additionally, toxicities of these drugs individually and in combination will need to be considered thoroughly, especially when toxicities affect organs injured in the disease process of COVID-19.

## Conclusions

In summary, we have compiled literature of marketed drugs and supplements with preclinical and/or clinical data against coronaviruses along with pharmacokinetic data. We have identified several candidate small molecules with favorable pharmacokinetic properties for further evaluation for repurposing against SARS-CoV-2. This assembly may be beneficial for researchers comparing the vast amount of data available for the therapeutic potential of available drugs for further studies on treatments for COVID-19.

## Supplementary Material

mjab002_Supplementary_Data
