# Supplementary material for: Available drugs and supplements for rapid deployment for treatment of COVID-19
Source: J Mol Cell Biol. 2021 Jan 25;13(3):232–6. doi: 10.1093/jmcb/mjab002 (PMC7928750; doi:10.1093/jmcb/mjab002)
Supplement: mjab002_Supplementary_Data [file mjab002_supplementary_data.pdf]

Supplementary material

Supplementary Table S1 Extended data on available drugs or supplements acting on the viral life cycle with blood concentrations greater than 5× their IC50s against SARS-CoV-2.

**Drug or supplement name**	**Cmax or Ctrough in humans**	**Cmax or Ctrough in µM** (*if no citation, calculated from corresponding column 2 data)	**Cytotoxicity or SI in µM (cell type)** (*corresponding to experiments in the reference in column 5)	**Cell culture data IC50 in µM**	**Animal data**	**Human data in coronavirus patients**	**Potential mechanism of action as an anti-viral reagent**
Baicalin	Cmax=74 µg/ml (360 mg IV in human subjects)[1]	Cmax=165.77	CC50>100 µg/ml; SI>4–8 (fRhK4 cells)[1]	**SARS-CoV-2** 3CLpro inhibition IC50=6.41[2]<br><br>**SARS-CoV-1** IC50=12.5–25 µg/ml (28–56 µM) (fRhK4 cells)[1]	NA	NA	Inhibits SARS-CoV-2 3CLpro *in vitro*[2]<br><br>Predicted to bind to SARS-CoV-2 PLpro and RDRP (*in silico*)[3]<br><br>Predicted to bind to 3CLpro and furin (*in silico*)[4]
Dalbavancin	Cmax=312 mg/L (1120 mg 30 min infusion in healthy subjects)[5]	Cmax=171.74		**SARS-CoV-1** IC50 ~0.053 (HEK 293/hACE2)[6]<br><br>**MERS** IC50=2.99; **SARS-CoV-1** IC50=9.64 (HEK 293T)[7]	SARS-CoV-2 mouse model treated with dalbavancin (130 mg/kg intraperitoneal day 0) and SARS-CoV-2 rhesus macaque model treated with dalbavancin (60 mg/kg day 0 and 30 mg/kg day 4 by phleboclysis) showed lower viral load and reduced histopathological injury compared to control[6]	NA	Binds to ACE2 and inhibits interaction with SARS-CoV-2 SPIKE[6]<br><br>Inhibits SARS-CoV-1 and MERS entry, perhaps through cathepsin L in the late endosome[7]

Dipyridamole	Cmax=475.25 or 498.1 ng/ml, depending on formulation (25 mg in healthy volunteers)[8]	Cmax=0.94 or 0.99		**SARS-CoV-2** IC50<0.1 (Vero E6 cells)[9]<br><br>**SARS-CoV-2** 3CLpro IC50=0.53[9]<br><br>No effect on **HCoV-NL63**[10]	NA	Prospective, open-label, randomized, controlled study of COVID-19 patients showed those receiving dipyridamole treatment (50 mg 3×/day) had higher clinical remission rates, decreased D-dimer, and increased lymphocytes and platelets compared to control patients[9].	Inhibits 3CLpro and viral entry and may have anti-inflammation, anti-fibrotic, and anti-coagulation activities [9]
Eltrombopag	Cmax=24.8 µg/ml (200 mg/day for 5 days in healthy subjects)[11]	Cmax=56.05	CC50>50; SI=6.05 (Vero cells)[12]	**SARS-CoV-2** IC50=8.27 (Vero cells)[12]<br><br>**SARS-CoV-2** IC50=8.38 (Calu-3 cells)[13]	NA	NA	Predicted to bind to SARS-CoV-2 RDRP (*in silico*)[14]<br><br>Binds to SARS-CoV-2 SPIKE protein and may destabilize the interaction between SPIKE and ACE2[15]<br><br>Predicted to bind to SARS-CoV-1 RDRP-NSP7 and RDRP-NSP8 interfaces (*in silico*)[16]

Favipiravir	Cmax=61.50 mg/L (600 mg 2×/day in healthy subjects)[17]	Cmax=391.4	CC50>400; SI>6.46 (Vero E6 cells)[18]<br><br>CC50>500; SI=1 (Vero cells)[12]<br><br>CC50>100 (Vero E6 cells)[19]	**SARS-CoV-2** IC50=61.88 (Vero E6 cells)[18]<br><br>**SARS-CoV-2** IC50>500 (Vero cells)[12]<br><br>**SARS-CoV-2** IC50>100 (Vero E6 cells)[19]<br><br>**SARS-CoV-2** IC50=207.1 (replication), 118.3 (CPE) (Vero E6 cells)[20]<br><br>**SARS-CoV-2** IC50>100 (Vero E6 cells)[21]	SARS-CoV-2 Syrian hamster model treated with favipiravir (1200 mg/kg day 1, then 1000 mg/kg/day) had lower amounts of infectious virus in the lung, improved lung histopathology, and reduced viral transmission compared to control[22]	Prospective, open-label, randomized, controlled study of COVID-19 patients showed those receiving favipiravir (1600 mg or 2200 mg on day 1, then 600 mg 3×/day) had no significant reduction in viral load compared to control[21].<br><br>Prospective, open-label, randomized, controlled study of COVID-19 patients receiving favipiravir (1800 mg 2×/day on day 1, then 800 mg 2×/day) showed shorter time to clinical improvement and cessation of viral shedding compared to control[23].<br><br>Prospective, open-label, non-randomized, before–after controlled study of COVID-19 patients showed those treated with **favipiravir** (1600 mg 2×/day on day 1, then 600 mg 2×/day) **+ IFNα (5 million U 2×/day)** had improved chest imaging and shorter time to viral clearance compared to control[24].<br><br>Prospective, randomized study of COVID-19 patients treated with favipiravir (1800 mg 2×/day on day 1, then 800 mg 2×/day) in early stage compared to late stage disease[25]<br><br>Prospective, uncontrolled study of COVID-19 patients requiring mechanical ventilation receiving favipiravir (3600 mg for day 1, then 1600 mg)[26]<br><br>Case reports of COVID-19 patients treated with favipiravir[27-33]	Causes lethal mutagenesis of SARS-CoV-2 through incorporation into RDRP[20]

Mycophenolate mofetil	Cmax of mycophenolic acid, metabolite of mycophenolate mofetile, ~25 mg/L (1 g mycophenolate mofetil in healthy subjects)[34]	Mycophenolic acid Cmax=78.04	Mycophenolate acid CC50 in various cell lines ~3.5[35]<br><br>Mycophenolic acid CC50>100 (Vero E6 cells)[36]<br><br>Mycophenolic acid CC50>32; SI>195 (Vero cells)[37]	Mycophenolic acid: **SARS-CoV-2** IC50=0.87 (Vero/TMPRSS2 cells)[38]<br><br>**SARS-CoV-2** IC50=0.101 (Vero E6 cells)[36]<br><br>**SARS-CoV-1** IC50>50 mg/L (>156.08 µM) (Vero cells)[39]<br><br>**MERS** IC50=0.17 (Vero cells)[37]<br><br>**MERS** IC50=2.87 (Vero E6 cells)[40]<br><br>**HCoV-OC43** IC50=1.95; **HCoV-NL63** IC50=0.18; **MERS** IC50=1.95; **MHV-A59** IC50=0.17 (various cell lines)[35]<br><br>**MERS** PLpro IC50=222.5-247.6, **synergistic effect with 6-mercaptopurine;** no effect on **SARS-CoV-1** PLpro[41]	SARS-CoV-1 mouse model treated with mycophenolic acid (75 and 10 mg/kg) had slight increase in viral titer compared to control[42]<br><br>MERS marmoset model treated with mycophenolate mofetil (12 mg/kg) may have increased viral load and severity of disease compared to no treatment, lopinavir+ritonavir, or IFN1b[43]	NA	

Nafamostat	Cmax=60.43 ng/ml (40 mg IV in healthy subjects)[44]	Cmax=0.17	CC50>100; SI>4.44 (Vero E6 cells)[18]	**SARS-CoV-2** IC50=0.0022 (Calu-3 cells), 13.88 (Vero cells)[13]<br><br>**SARS-CoV-2** IC50=22.5 (Vero E6 cells)[18]<br><br>**SARS-CoV-2** IC50=0.01, 0.007 (MOI 0.1, 0.01) (Calu3 cells); IC50=31.6 (Vero E6/TMPRSS2 cells); >100 with no pretreatment[45]<br><br>**SARS-CoV-2** IC50=0.005; **SARS-CoV-1** IC50=0.001; **MERS** IC50=0.006 (Calu-3 cells)[46]<br><br>**MERS** IC50 ~0.001 (Calu3 cells), 0.1 (HEK 293FT cells)[47]	NA	Case reports of COVID-19 patients treated with nafamostat[31, 32, 48, 49]	Inhibits MERS entry through inhibition of TMPRSS2[47]
Nitazoxanide	Nitazoxanide as a parent compound is not detectable in plasma, which is rapidly metabolized to its active metabolites tizoxanide and tizoxanide glucuronide. Cmax of tizoxanide ~10.73 µg/ml (500 mg in healthy subjects)[50]	Tizoxanide Cmax ~39.09	CC50>35.53; SI>16.76 (Vero E6 cells)[18]<br><br>SI>50 (A72 cells)[51, 52]	**SARS-CoV-2** IC50=2.12 (Vero E6 cells)[18]<br><br>Tizoxanide: **Canine coronavirus** IC50=1 µg/ml (3.77 µM) (A72 cells)[51, 52]<br><br>**MHV-2aFLS** IC50=1 (DBT cells)[53]	NA	Prospective, randomized, controlled trial of patients with influenza-like illness. Those with coronaviruses (NL63, 229E, OC43) showed no statistically significant difference in days to hospital discharge when treated with nitazoxanide compared to control[54].	Inhibits MHV when applied either pre- or post-infection[53]

Remdesivir		Cmax=9 (200 mg IV in healthy subjects)[55]	No significant cytotoxicity in Vero E6 or Calu3 cells; no significant cytotoxicity at <10 µM in primary human lung and airway epithelial cells[56]<br><br>CC50>100; SI>129.87 (Vero cells)[18]<br><br>CC50>25, >50; SI=2.19, 6.07; CC50>50; SI=6.07 (immunofluorescence, CPE) (Vero cells)[12]<br><br>CC50>100 (Vero E6 cells)[19]	**SARS-CoV-2** IC50=1.65 (Vero E6 cells), 0.28 (Calu3 cells), 0.01(primary human lung and airway epithelial cells)[56]<br><br>**SARS-CoV-2** IC50=0.77 *in vitro* (Vero E6 cells)[18]<br><br>**SARS-CoV-2** IC50=11.41(immunofluorescence), 8.24 (CPE) (Vero cells)[12]<br><br>**SARS-CoV-2** IC50=1.67 (Vero E6 cells)[57]<br><br>**SARS-CoV-2** IC50=23.15 (Vero E6 cells), **synergy with emetine**[19]<br><br>**SARS-CoV-2** IC50=1.3 (Calu-3 cells)[13]	SARS-CoV-2 rhesus macaque model treated with remdesivir (10 mg/kg loading dose, then 5 mg/kg per day IV) showed reduced viral load and lung damage compared to control[58]<br><br>SARS-CoV-2 mouse model treated with remdesivir (25 mg/kg) showed more pulmonary function and reduced viral titer compared to control[56]<br><br>MERS rhesus macaque models treated with remdesivir (IV dose of 5 mg/kg) prophylactically or for treatment showed improved clinical signs and reduced viral load compared to vehicle control[59]	Prospective, randomized, controlled trial of COVID-19 patients showed those receiving remdesivir (200 mg IV day 1, then 100 mg/day) had faster time to clinical improvement, but not statistically significantly different compared to control[60].<br><br>Prospective, randomized, controlled study of COVID-19 patients showed those treated with remdesivir (200 mg IV day 1, then 100 mg/day) had shorter time to recovery compared to control[61].<br><br>Prospective, open-label, randomized, controlled study of hospitalized COVID-19 patients showed those treated with remdesivir (200 mg IV day 1, then 100 mg/day) for 5 days, but not 10 days, had significantly improved clinical status on day 11 compared to control[62].<br><br>Prospective, open-label, randomized study of hospitalized patients with COVID-19 treated with remdesivir (200 mg IV day 1, then 100 mg/day) showed no difference in clinical status at day 14 between those treated for 5 or 10 days[63].<br><br>Retrospective, controlled study of hospitalized COVID-19 patients on mechanical ventilation showed those treated with remdesivir (200 mg IV day 1, then 100 mg/day) had higher survival rates compared to control[64].<br><br>Retrospective, case-controlled study of COVID-19 patients on mechanical ventilation showed those treated with remdesivir (200 mg IV day 1, then 100 mg/day) had higher rate of hospital discharge and extubation, but not lower risk of death, compared to control[65].<br><br>COVID-19 patients treated compassionately with remdesivir (200 mg IV day 1, then 100 mg/day)[66, 67]<br><br>Case reports of COVID-19 patients treated with remdesivir[68,69, 70,71,72, 73,74]	Inhibits SARS-CoV-2 RDRP (*in vitro*)[75]<br><br>Inhibits SARS-CoV-1/SARS-CoV-2 RDRP (*in vitro*)[56]<br><br>Binds to SARS-CoV-2 RDRP *in vitro*[76]<br><br>Inhibits SARS-CoV-2 post-viral entry[18]<br><br>Predicted to bind to SARS-CoV-2 RDRP (*in silico*)[77,56, 78 79, 80]<br><br>Predicted to bind to SARS-CoV-2 3CLpro (*in silico*)[81, 82]<br><br>Predicted to bind to SARS-CoV-2 Nsp1 (*in silico*)[83]

Sulfadoxine	Cmax=165.15–183.07 mg/L (1500 mg sulfadoxine, 75 mg pyrimethamine in healthy volunteers)[84]	Cmax=532.17–589.89	CC50>40; SI>1.13 (Vero E6 cells)[57]	**SARS-CoV-2** IC50=35.37 (Vero E6 cells)[57]	NA	NA	
Teicoplanin	Ctrough plasma =14.5–21.8 mg/L (from pooled hospital data of patients treated with teicoplanin)[85]	Ctrough plasma =7.71–11.59	CC50>500 (HEK 293T cells)[7]	**SARS-CoV-1** IC50=3.76<br><br>**MERS** IC50=0.63 (HEK 293T cells)[7]<br><br>**SARS-CoV-2** 3CLpro inhibition IC50 ~1.5[86]	NA	Retrospective, uncontrolled study of hospitalized COVID-19 patients receiving teicoplanin (600 mg/day)[87]	In SARS-CoV-1 and MERS, inhibits viral entry, perhaps through cathepsin L in the late endosome[7]<br><br>Predicted to bind to SARS-CoV-2 Nsp3, Nsp15, Nsp9 (*in silico*)[88]

CC50, half maximal cytotoxic concentration; Cmax, maximum plasma or blood concentration; CPE, cytopathic effect; Ctrough, lowest concentration before next dose; HCoV-NL63, human coronavirus NL63; HCoV-O463, human coronavirus O463; IC50, half maximal inhibitory concentration; IFN, interferon; IV, intravenous; MERS, Middle East respiratory syndrome; MHV, mouse hepatitis virus; MOI, multiplicity of infection; Nsp, nonstructural protein; PLpro, papain-like protease; RDRP, RNA-dependent RNA polymerase; SARS-CoV-1, severe acute respiratory syndrome coronavirus 1; SARS-CoV-2, severe acute respiratory syndrome coronavirus 2; SI, selectivity index; 3CLpro, coronavirus main proteinase.

Supplementary Table S2 Available drugs or supplements acting on the viral life cycle of non-SARS-CoV-2 coronaviruses with blood concentrations greater than 5 × their IC50s.

**Drug or supplement name**	**Cmax in humans**	**Cmax in µM** (* if no citation, calculated from corresponding column 2 data)	**Cytotoxicity or SI in µM (cell type)** (*corresponding to experiments in the reference in column 5)	**Cell culture data IC50 in µM**	**Animal data**	**Human data in coronavirus patients**	**Potential mechanism of action as an anti-viral reagent**
Oxaprozin	Cmax=286 µg/ml (1200 mg/day, 7 days in healthy volunteers)[89]	Cmax=975.05		Inhibits >75% **MHV** infection when tested at 10 µM (DBT cells)[53]	NA	NA	Predicted to bind to SARS-CoV-2 SPIKE protein (*in silico*)[90]<br><br>Predicted to bind to SARS-CoV-2 3CLpro (*in silico*)[91]<br><br>May inhibit MHV entry as inhibitory effect seen by time of addition studies but increased luciferase signal if added 3 h post-infection[53]
Telavancin	Cmax=105 µg/ml (10 mg/kg IV infusion in healthy controls)[92]	Cmax=59.8		**MERS** IC50=3.24; **SARS-CoV-1** IC50=3.45 (HEK 293T cells)[7]	NA	NA	In SARS-CoV-1 and MERS, inhibits viral entry, perhaps through cathepsin L in the late endosome[7]

Cmax, maximum plasma or blood concentration; CPE, cytopathic effect; IC50, half maximal inhibitory concentration; IV, intravenous; MERS, Middle East respiratory syndrome; MHV, mouse hepatitis virus; SARS-CoV-1, severe acute respiratory syndrome coronavirus 1; SARS-CoV-2, severe acute respiratory syndrome coronavirus 2; 3CLpro, coronavirus main proteinase.

Supplementary Table S3 Commentary on drugs or supplements acting on the viral life cycle of SARS-CoV-2 with blood concentrations greater than 5 × their IC50s.

Drug or supplement name	Indications, side effects, and comments
Baicalin	Baicalin is marketed as an oral supplement and therefore is not approved for the treatment of any specific disease. It is a flavonoid with antiviral, anti-oxidative, anti-inflammatory, and anti-proliferative activities[93]. Its side effect profile has not been carefully evaluated.
Dalbavancin	Dalbavancin is an IV medication approved for acute gram-positive bacterial skin and skin structure infections. Common side effects include nausea, headache, diarrhea, vomiting, pruritis, and rash[94].
Dipyridamole	Dipyridamole is an oral or IV medication approved as an anticoagulation agent after heart valve replacement surgery. Common side effects include angina, ST segment depression, facial flushing, and ischemia, though some of these symptoms of coronary steal may be reversed with aminophylline administration, as well as headache, dizziness, lightheadedness, paresthesia, nausea, and vomiting[95].
Eltrombopag	Eltrombopag is an oral medication approved for thrombocytopenia and aplastic anemia. Common side effects include deep vein thrombosis, palpitations, prolonged QT interval, thrombotic events, abnormal hepatic function or failure, increased liver enzymes, increased bilirubin, anemia, neutropenia, decreased hemoglobin, lymphopenia, diarrhea, nausea, vomiting, gastrointestinal upset, pruritis, rash, alopecia, eczema, dry skin, erythema, hyperhidrosis, night sweats, urinary tract infection, influenza, decreased appetite, hyperglycemia, decreased albumin, myalgia, muscle spasm, arthralgia, headache, dizziness, hepatic encephalopathy, lethargy, paresthesia, cataract development, retinal hemorrhage, dry eye, itchy eye, malignant hepatic neoplasm, insomnia, anxiety, depression, irritability, cough, upper respiratory infection, nasopharyngitis, rhinorrhea, dyspnea, and cytogenic abnormalities[96].
Favipiravir	Favipiravir is an oral medication approved in Japan for influenza. Common side effects include hyperuricemia, diarrhea, increased triglycerides, increased liver enzymes, and reduced neutrophil count[97].
Mycophenolate mofetil	Mycophenolate mofetil is an oral or IV medication approved for prevention of transplant organ rejection. Common side effects include diarrhea, nausea, vomiting, abdominal pain, gastroenteritis, gastrointestinal infection, leukopenia, anemia, sepsis, lymphoma, hematuria, kidney tubular necrosis, renal impairment, edema, hyperphosphatemia, hyperkalemia, hyperglycemia, dyspnea, respiratory tract infections, cough, fungal skin infection, skin hypertrophy, rash, alopecia, arthralgia, malaise, hepatitis, tachycardia, hypotension, hypertension, vasodilation, and urinary tract infection[98]. Of note, mycophenolate mofetil is rapidly metabolized to mycophenolic acid.
Nafamostat	Nafamostat is an IV medication approved in Japan for pancreatitis. Common side effects may include hyperkalemia[99].
Nitazoxanide	Nitazoxanide is an oral medication approved for diarrhea from parasite infections. Common side effects include nausea, abdominal pain, headache, chromaturia[100]. Of note, nitazoxanide is rapidly metabolized to tizoxanide.
Remdesivir	Remdesivir is an IV medication FDA approved for emergency use for treatment of COVID-19. Common side effects are not yet established. Of note, remdesivir is rapidly converted to metabolites.
Sulfadoxine	Sulfadoxine is an oral medication approved for the treatment of malaria and is often formulated with pyrimethamine. Common side effects include leukopenia, anorexia, diarrhea, abdominal pain, vomiting, hepatitis, cough, and headache[101].
Teicoplanin	Teicoplanin is an IV, intramuscular, or oral medication approved in several countries outside of USA for the treatment of gram-positive bacterial infections. Common side effects include rash, pruritis, erythema, pain, and fever[102].

References

1. Chen F, Chan KH, Jiang Y, et al. In vitro susceptibility of 10 clinical isolates of SARS coronavirus to selected antiviral compounds. *Journal of Clinical Virology.* 2004/09/01/ 2004;31(1):69-75.
2. Su H-x, Yao S, Zhao W-f, et al. Anti-SARS-CoV-2 activities in vitro of Shuanghuanglian preparations and bioactive ingredients. *Acta Pharmacologica Sinica.* 2020/09/01 2020;41(9):1167-1177.
3. Wu C, Liu Y, Yang Y, et al. Analysis of therapeutic targets for SARS-CoV-2 and discovery of potential drugs by computational methods. *Acta Pharm Sin B.* May 2020;10(5):766-788.
4. Manikyam HK, Joshi SK. Whole Genome Analysis and Targeted Drug Discovery Using Computational Methods and High Throughput Screening Tools for Emerged Novel Coronavirus (2019-nCoV). *J Pharm Drug Res.* 2020;3(2):341-361.
5. Leighton A, Gottlieb AB, Dorr MB, et al. Tolerability, pharmacokinetics, and serum bactericidal activity of intravenous dalbavancin in healthy volunteers. *Antimicrobial agents and chemotherapy.* 2004;48(3):940-945.
6. Wang G, Yang M-L, Duan Z-L, et al. Dalbavancin binds ACE2 to block its interaction with SARS-CoV-2 spike protein and is effective in inhibiting SARS-CoV-2 infection in animal models. *Cell Research.* 2020/12/01 2020.
7. Zhou N, Pan T, Zhang J, et al. Glycopeptide antibiotics potently inhibit cathepsin L in the late endosome/lysosome and block the entry of Ebola virus, Middle East respiratory syndrome coronavirus (MERS-CoV), and severe acute respiratory syndrome coronavirus (SARS-CoV). *Journal of Biological Chemistry.* 2016;291(17):9218-9232.
8. Beiki D, Amini M, Dowlatabadi R, Pirali M. Comparative bioavailability of two tablet formulations of dipyridamole in healthy volunteers. *Iranian Journal of Pharmaceutical Research.* 2010:213-216.
9. Liu X, Li Z, Liu S, et al. Potential therapeutic effects of dipyridamole in the severely ill patients with COVID-19. *Acta pharmaceutica Sinica. B.* 2020;10(7):1205-1215.
10. Pyrc K, Bosch BJ, Berkhout B, et al. Inhibition of Human Coronavirus NL63 Infection at Early Stages of the Replication Cycle. *Antimicrobial Agents and Chemotherapy.* 2006;50(6):2000-2008.
11. Matthys G, Park JW, McGuire S, et al. Clinical pharmacokinetics, platelet response, and safety of eltrombopag at supratherapeutic doses of up to 200 mg once daily in healthy volunteers. *Journal of clinical pharmacology.* Mar 2011;51(3):301-308.
12. Jeon S, Ko M, Lee J, et al. Identification of Antiviral Drug Candidates against SARS-CoV-2 from FDA-Approved Drugs. *Antimicrobial Agents and Chemotherapy.* 2020;64(7):e00819-00820.
13. Ko M, Jeon S, Ryu W-S, Kim S. Comparative analysis of antiviral efficacy of FDA-approved drugs against SARS-CoV-2 in human lung cells. *Journal of Medical Virology.*
14. Gul S, Ozcan O, Asar S, Okyar A, Barıs I, Kavakli IH. In silico identification of widely used and well-tolerated drugs as potential SARS-CoV-2 3C-like protease and viral RNA-dependent RNA polymerase inhibitors for direct use in clinical trials. *J Biomol Struct Dyn.* Aug 5 2020:1-20.
15. Feng S, Luan X, Wang Y, et al. Eltrombopag is a potential target for drug intervention in SARS-CoV-2 spike protein. *Infection, Genetics and Evolution.* 2020/11/01/ 2020;85:104419.
16. Ruan Z, Liu C, Guo Y, et al. SARS-CoV-2 and SARS-CoV: Virtual screening of potential inhibitors targeting RNA-dependent RNA polymerase activity (NSP12). *Journal of Medical Virology.*

17. Madelain V, Nguyen THT, Olivo A, et al. Ebola Virus Infection: Review of the Pharmacokinetic and Pharmacodynamic Properties of Drugs Considered for Testing in Human Efficacy Trials. *Clinical pharmacokinetics.* 2016;55(8):907-923.
18. Wang M, Cao R, Zhang L, et al. Remdesivir and chloroquine effectively inhibit the recently emerged novel coronavirus (2019-nCoV) in vitro. *Cell Research.* 2020/03/01 2020;30(3):269-271.
19. Choy K-T, Wong AY-L, Kaewpreedee P, et al. Remdesivir, lopinavir, emetine, and homoharringtonine inhibit SARS-CoV-2 replication in vitro. *Antiviral research.* 2020/06/01/ 2020;178:104786.
20. Shannon A, Selisko B, Le N-T-T, et al. Rapid incorporation of Favipiravir by the fast and permissive viral RNA polymerase complex results in SARS-CoV-2 lethal mutagenesis. *Nature communications.* 2020/09/17 2020;11(1):4682.
21. Lou Y, Liu L, Yao H, et al. Clinical Outcomes and Plasma Concentrations of Baloxavir Marboxil and Favipiravir in COVID-19 Patients: An Exploratory Randomized, Controlled Trial. *European Journal of Pharmaceutical Sciences.* 2020/10/25/ 2020:105631.
22. Kaptein SJF, Jacobs S, Langendries L, et al. Favipiravir at high doses has potent antiviral activity in SARS-CoV-2−infected hamsters, whereas hydroxychloroquine lacks activity. *Proceedings of the National Academy of Sciences.* 2020;117(43):26955-26965.
23. Udwadia ZF, Singh P, Barkate H, et al. Efficacy and Safety of Favipiravir, an Oral RNA-Dependent RNA Polymerase Inhibitor, in Mild-to-Moderate COVID-19: A Randomized, Comparative, Open-Label, Multicenter, Phase 3 Clinical Trial. *International Journal of Infectious Diseases.* 2020/11/16/ 2020.
24. Cai Q, Yang M, Liu D, et al. Experimental Treatment with Favipiravir for COVID-19: An Open-Label Control Study. *Engineering.* 2020/03/18/ 2020.
25. Doi Y, Hibino M, Hase R, et al. A Prospective, Randomized, Open-Label Trial of Early versus Late Favipiravir Therapy in Hospitalized Patients with COVID-19. *Antimicrobial Agents and Chemotherapy.* 2020;64(12):e01897-01820.
26. Yamamura H, Matsuura H, Nakagawa J, Fukuoka H, Domi H, Chujoh S. Effect of favipiravir and an anti-inflammatory strategy for COVID-19. *Critical Care.* 2020/07/09 2020;24(1):413.
27. Takahashi H, Iwasaki Y, Watanabe T, et al. Case studies of SARS-CoV-2 treated with favipiravir among patients in critical or severe condition. *International Journal of Infectious Diseases.* 2020/11/01/ 2020;100:283-285.
28. Murohashi K, Hagiwara E, Kitayama T, et al. Outcome of early-stage combination treatment with favipiravir and methylprednisolone for severe COVID-19 pneumonia: A report of 11 cases. *Respiratory Investigation.* 2020/08/28/ 2020.
29. Inoue H, Jinno M, Ohta S, et al. Combination treatment of short-course systemic corticosteroid and favipiravir in a successfully treated case of critically ill COVID-19 pneumonia with COPD. *Respiratory Medicine Case Reports.* 2020/01/01/ 2020;31:101200.
30. Fu D, Cao R, Zhao L, Li W, Zhong W, Wen J. Oral favipiravir for patients with delayed SARS-CoV-2 viral RNA clearance: a case series. *Critical Care.* 2020/09/25 2020;24(1):578.
31. Doi K, Ikeda M, Hayase N, et al. Nafamostat mesylate treatment in combination with favipiravir for patients critically ill with Covid-19: a case series. *Critical Care.* 2020/07/03 2020;24(1):392.
32. Osawa I, Okamoto K, Ikeda M, et al. Dynamic changes in fibrinogen and D-dimer levels in COVID-19 patients on nafamostat mesylate. *Journal of Thrombosis and Thrombolysis.* 2020/09/12 2020.
33. Takoi H, Togashi Y, Fujimori D, et al. Favipiravir-induced fever in coronavirus disease 2019: A report of two cases. *International Journal of Infectious Diseases.* 2020/09/28/ 2020.

34. Bullingham RES, Nicholls AJ, Kamm BR. Clinical Pharmacokinetics of Mycophenolate Mofetil. *Clinical Pharmacokinetics.* 1998/06/01 1998;34(6):429-455.

35. Shen L, Niu J, Wang C, et al. High-Throughput Screening and Identification of Potent Broad-Spectrum Inhibitors of Coronaviruses. *Journal of virology.* Jun 15 2019;93(12).

36. Wan W, Zhu S, Li S, et al. High-Throughput Screening of an FDA-Approved Drug Library Identifies Inhibitors against Arenaviruses and SARS-CoV-2. *ACS infectious diseases.* 2020.

37. Chan JF, Chan KH, Kao RY, et al. Broad-spectrum antivirals for the emerging Middle East respiratory syndrome coronavirus. *The Journal of infection.* Dec 2013;67(6):606-616.

38. Kato F, Matsuyama S, Kawase M, Hishiki T, Katoh H, Takeda M. Antiviral activities of mycophenolic acid and IMD-0354 against SARS-CoV-2. *Microbiology and Immunology.* 2020;64(9):635-639.

39. Cinatl J, Morgenstern B, Bauer G, Chandra P, Rabenau H, Doerr HW. Glycyrrhizin, an active component of liquorice roots, and replication of SARS-associated coronavirus. *Lancet.* 2003;361(9374):2045-2046.

40. Hart BJ, Dyall J, Postnikova E, et al. Interferon-β and mycophenolic acid are potent inhibitors of Middle East respiratory syndrome coronavirus in cell-based assays. *J Gen Virol.* 2014;95(Pt 3):571-577.

41. Cheng KW, Cheng SC, Chen WY, et al. Thiopurine analogs and mycophenolic acid synergistically inhibit the papain-like protease of Middle East respiratory syndrome coronavirus. *Antiviral research.* Mar 2015;115:9-16.

42. Barnard DL, Day CW, Bailey K, et al. Enhancement of the infectivity of SARS-CoV in BALB/c mice by IMP dehydrogenase inhibitors, including ribavirin. *Antiviral research.* 2006;71(1):53-63.

43. Chan JF-W, Yao Y, Yeung M-L, et al. Treatment With Lopinavir/Ritonavir or Interferon-β1b Improves Outcome of MERS-CoV Infection in a Nonhuman Primate Model of Common Marmoset. *The Journal of infectious diseases.* 2015;212(12):1904-1913.

44. Cao YG, Zhang M, Yu D, Shao JP, Chen YC, Liu XQ. A method for quantifying the unstable and highly polar drug nafamostat mesilate in human plasma with optimized solid-phase extraction and ESI-MS detection: more accurate evaluation for pharmacokinetic study. *Analytical and bioanalytical chemistry.* Jun 2008;391(3):1063-1071.

45. Yamamoto M, Kiso M, Sakai-Tagawa Y, et al. The Anticoagulant Nafamostat Potently Inhibits SARS-CoV-2 S Protein-Mediated Fusion in a Cell Fusion Assay System and Viral Infection In Vitro in a Cell-Type-Dependent Manner. *Viruses.* 2020;12(6):629.

46. Hoffmann M, Schroeder S, Kleine-Weber H, Müller MA, Drosten C, Pöhlmann S. Nafamostat mesylate blocks activation of SARS-CoV-2: New treatment option for COVID-19. *Antimicrobial Agents and Chemotherapy.* 2020:AAC.00754-00720.

47. Yamamoto M, Matsuyama S, Li X, et al. Identification of Nafamostat as a Potent Inhibitor of Middle East Respiratory Syndrome Coronavirus S Protein-Mediated Membrane Fusion Using the Split-Protein-Based Cell-Cell Fusion Assay. *Antimicrobial Agents and Chemotherapy.* 2016;60(11):6532-6539.

48. Doi S, Akashi YJ, Takita M, et al. Preventing thrombosis in a COVID-19 patient by combinatorial therapy with nafamostat and heparin during extracorporeal membrane oxygenation. *Acute Medicine & Surgery.*n/a(n/a).

49. Jang S, Rhee J-Y. Three cases of treatment with nafamostat in elderly patients with COVID-19 pneumonia who need oxygen therapy. *International Journal of Infectious Diseases.* 2020/07/01/ 2020;96:500-502.

50. Balderas-Acata J, Ríos-Rogríguez Bueno E, Perez-Becerril F, Espinosa-Martinez C, Burke-Fraga V, la Parra M. Bioavailability of two oral-suspension formulations of a single dose of nitazoxanide 500 mg: an open-label, randomized-sequence, two-period crossover, comparison in healthy fasted mexican adult volunteers. *J Bioequiv Availab.* 2011;3:43-47.

51. Rossignol J, Santoro M. Activity of thiazolides against other respiratory viruses than influenza. *Influenza and Other Respiratory Virus Infections: Advances in Clinical Management.* 2014.

52. Rossignol J-F. Nitazoxanide: A first-in-class broad-spectrum antiviral agent. *Antiviral research.* 2014/10/01/ 2014;110:94-103.

53. Cao J, Forrest JC, Zhang X. A screen of the NIH Clinical Collection small molecule library identifies potential anti-coronavirus drugs. *Antiviral research.* 2015/02/01/ 2015;114:1-10.

54. Gamiño-Arroyo AE, Guerrero ML, McCarthy S, et al. Efficacy and Safety of Nitazoxanide in Addition to Standard of Care for the Treatment of Severe Acute Respiratory Illness. *Clinical Infectious Diseases.* 2019;69(11):1903-1911.

55. Agency EM. Summary on Compassionate Use. EMA/178637/2020 – Rev.2 ed2020.

56. Pruijssers AJ, George AS, Schäfer A, et al. Remdesivir Inhibits SARS-CoV-2 in Human Lung Cells and Chimeric SARS-CoV Expressing the SARS-CoV-2 RNA Polymerase in Mice. *Cell Reports.* 2020/07/21/ 2020;32(3):107940.

57. Touret F, Gilles M, Barral K, et al. In vitro screening of a FDA approved chemical library reveals potential inhibitors of SARS-CoV-2 replication. *Scientific Reports.* 2020/08/04 2020;10(1):13093.

58. Williamson BN, Feldmann F, Schwarz B, et al. Clinical benefit of remdesivir in rhesus macaques infected with SARS-CoV-2. *Nature.* 2020/09/01 2020;585(7824):273-276.

59. de Wit E, Feldmann F, Cronin J, et al. Prophylactic and therapeutic remdesivir (GS-5734) treatment in the rhesus macaque model of MERS-CoV infection. *Proceedings of the National Academy of Sciences.* 2020:201922083.

60. Wang Y, Zhang D, Du G, et al. Remdesivir in adults with severe COVID-19: a randomised, double-blind, placebo-controlled, multicentre trial. *The Lancet.*

61. Beigel JH, Tomashek KM, Dodd LE, et al. Remdesivir for the Treatment of Covid-19 — Final Report. *New England Journal of Medicine.* 2020;383(19):1813-1826.

62. Spinner CD, Gottlieb RL, Criner GJ, et al. Effect of Remdesivir vs Standard Care on Clinical Status at 11 Days in Patients With Moderate COVID-19: A Randomized Clinical Trial. *Jama.* 2020;324(11):1048-1057.

63. Goldman JD, Lye DCB, Hui DS, et al. Remdesivir for 5 or 10 Days in Patients with Severe Covid-19. *New England Journal of Medicine.* 2020.

64. Pasquini Z, Montalti R, Temperoni C, et al. Effectiveness of remdesivir in patients with COVID-19 under mechanical ventilation in an Italian ICU. *The Journal of antimicrobial chemotherapy.* 2020:dkaa321.

65. Lapadula G, Bernasconi DP, Bellani G, et al. Remdesivir Use in Patients Requiring Mechanical Ventilation due to COVID-19. *Open Forum Infectious Diseases.* 2020;7(11).

66. Grein J, Ohmagari N, Shin D, et al. Compassionate Use of Remdesivir for Patients with Severe Covid-19. *New England Journal of Medicine.* 2020.

67. Antinori S, Cossu MV, Ridolfo AL, et al. Compassionate remdesivir treatment of severe Covid-19 pneumonia in intensive care unit (ICU) and Non-ICU patients: Clinical outcome and differences in post_treatment hospitalisation status. *Pharmacological Research.* 2020/05/11/ 2020:104899.

68. Holshue ML, DeBolt C, Lindquist S, et al. First Case of 2019 Novel Coronavirus in the United States. *New England Journal of Medicine.* 2020;382(10):929-936.
69. Lee C, Ahn MY, Byeon K, et al. Clinical Experience with Use of Remdesivir in the Treatment of Severe Acute Respiratory Syndrome Coronavirus 2: a Case Series. *Infection & chemotherapy.* 9/ 2020;52(3):369-380.
70. Dubert M, Visseaux B, Isernia V, et al. Case report study of the first five COVID-19 patients treated with remdesivir in France. *International Journal of Infectious Diseases.* 2020;98:290-293.
71. Hillaker E, Belfer JJ, Bondici A, Murad H, Dumkow LE. Delayed Initiation of Remdesivir in a COVID-19 Positive Patient. *Pharmacotherapy: The Journal of Human Pharmacology and Drug Therapy.*
72. Naqvi M, Zakowski P, Glucksman L, Smithson S, Burwick RM. Tocilizumab and Remdesivir in a Pregnant Patient With Coronavirus Disease 2019 (COVID-19). *Obstetrics & Gynecology.* 9000;Latest Articles.
73. Anderson J, Schauer J, Bryant S, Graves CR. The use of convalescent plasma therapy and remdesivir in the successful management of a critically ill obstetric patient with novel coronavirus 2019 infection: A case report. *Case Reports in Women's Health.* 2020/07/01/ 2020;27:e00221.
74. Frauenfelder C, Brierley J, Whittaker E, Perucca G, Bamford A. Infant With SARS-CoV-2 Infection Causing Severe Lung Disease Treated With Remdesivir. *Pediatrics.* 2020;146(3):e20201701.
75. Gordon CJ, Tchesnokov EP, Woolner E, et al. Remdesivir is a direct-acting antiviral that inhibits RNA-dependent RNA polymerase from severe acute respiratory syndrome coronavirus 2 with high potency. *Journal of Biological Chemistry.* April 13, 2020 2020.
76. Yin W, Mao C, Luan X, et al. Structural basis for inhibition of the RNA-dependent RNA polymerase from SARS-CoV-2 by remdesivir. *Science.* 2020:eabc1560.
77. Elfiky AA. Ribavirin, Remdesivir, Sofosbuvir, Galidesivir, and Tenofovir against SARS-CoV-2 RNA dependent RNA polymerase (RdRp): A molecular docking study. *Life Sciences.* 2020/07/15/ 2020;253:117592.
78. Koulgi S, Jani V, Uppuladinne MV, Sonavane U, Joshi R. Remdesivir-bound and ligand-free simulations reveal the probable mechanism of inhibiting the RNA dependent RNA polymerase of severe acute respiratory syndrome coronavirus 2. *RSC Advances.* 2020;10(45):26792-26803.
79. Shannon A, Le NT-T, Selisko B, et al. Remdesivir and SARS-CoV-2: Structural requirements at both nsp12 RdRp and nsp14 Exonuclease active-sites. *Antiviral research.* 2020/06/01/ 2020;178:104793.
80. Zhang L, Zhou R. Structural Basis of the Potential Binding Mechanism of Remdesivir to SARS-CoV-2 RNA-Dependent RNA Polymerase. *The Journal of Physical Chemistry B.* 2020/08/13 2020;124(32):6955-6962.
81. Hall DC, Ji H-F. A search for medications to treat COVID-19 via in silico molecular docking models of the SARS-CoV-2 spike glycoprotein and 3CL protease. *Travel Medicine and Infectious Disease.* 2020/04/12/ 2020:101646.
82. Naik VR, Munikumar M, Ramakrishna U, et al. Remdesivir (GS-5734) as a therapeutic option of 2019-nCOV main protease – in silico approach. *Journal of Biomolecular Structure and Dynamics.* 2020:1-14.
83. Sharma A, Tiwari V, Sowdhamini R. Computational search for potential COVID-19 drugs from FDA-approved drugs and small molecules of natural origin identifies several anti-virals and plant products. *Journal of Biosciences.* 2020/07/17 2020;45(1):100.

84. Liu Y-M, Zhang KE, Liu Y, et al. Pharmacokinetic Properties and Bioequivalence of Two Sulfadoxine/Pyrimethamine Fixed-Dose Combination Tablets: A Parallel-Design Study in Healthy Chinese Male Volunteers. *Clinical therapeutics.* 2012/11/01/ 2012;34(11):2212-2220.
85. Tobin CM, Lovering AM, Sweeney E, MacGowan AP. Analyses of teicoplanin concentrations from 1994 to 2006 from a UK assay service. *Journal of Antimicrobial Chemotherapy.* 2010;65(10):2155-2157.
86. Tripathi PK, Upadhyay S, Singh M, et al. Screening and evaluation of approved drugs as inhibitors of main protease of SARS-CoV-2. *International Journal of Biological Macromolecules.* 2020/12/01/ 2020;164:2622-2631.
87. Ceccarelli G, Alessandri F, d'Ettorre G, et al. Is teicoplanin a complementary treatment option for COVID-19? The question remains. *International journal of antimicrobial agents.* 2020;56(2):106029-106029.
88. Sadia A, Azam M, Basra M. Teicoplanin is a potential inhibitor of SARS CoV-2 replication enzymes: A docking study. *Asian Pacific Journal of Tropical Biomedicine.* December 1, 2020 2020;10(12):563-568.
89. Kahn SB, Hubsher JA. Effects of oxaprozin alone or in combination with aspirin on hemostasis and plasma protein binding. *Journal of clinical pharmacology.* Apr 1983;23(4):139-146.
90. Drew ED, Janes RW. Identification of a druggable binding pocket in the spike protein reveals a key site for existing drugs potentially capable of combating Covid-19 infectivity. *BMC Molecular and Cell Biology.* 2020/07/01 2020;21(1):49.
91. Mukundan Satyanarayanan DV. Ligands Based Drug Design for Covid 19-A Multi-Faceted Approach using Ligand Design, Molecular Docking and Binding Probability Calculation.
92. Goldberg MR, Wong SL, Shaw JP, Kitt MM, Barriere SL. Lack of effect of moderate hepatic impairment on the pharmacokinetics of telavancin. *Pharmacotherapy.* Jan 2010;30(1):35-42.
93. Zhao T, Tang H, Xie L, et al. Scutellaria baicalensis Georgi. (Lamiaceae): a review of its traditional uses, botany, phytochemistry, pharmacology and toxicology. *Journal of Pharmacy and Pharmacology.* 2019;71(9):1353-1369.
94. Drugs.com. Dalbavancin Side Effects. https://www.drugs.com/sfx/dalbavancin-side-effects.html. Accessed 13 October 2020.
95. Drugs.com. Dipyridamole Side Effects. https://www.drugs.com/sfx/dipyridamole-side-effects.html. Accessed 13 October 2020.
96. Drugs.com. Eltrombopag Side Effects. https://www.drugs.com/sfx/eltrombopag-side-effects.html. Accessed 13 October 2020.
97. Agrawal U, Raju R, Udwadia ZF. Favipiravir: A new and emerging antiviral option in COVID-19. *Med J Armed Forces India.* 2020:10.1016/j.mjafi.2020.1008.1004.
98. Drugs.com. Mycophenolate Mofetil Side Effects. https://www.drugs.com/sfx/mycophenolate-mofetil-side-effects.html. Accessed 12 October 2020.
99. Muto S, Imai M, Asano Y. Mechanisms of hyperkalemia caused by nafamostat mesilate. *Gen Pharmacol.* Dec 1995;26(8):1627-1632.
100. Drugs.com. Nitazoxanide Side Effects. https://www.drugs.com/sfx/nitazoxanide-side-effects.html. Accessed 12 October 2020.
101. Drugs.com. Pyrimethamine / sulfadoxine Side Effects. https://www.drugs.com/sfx/pyrimethamine-sulfadoxine-side-effects.html. Accessed 14 October 2020.
102. Drugs.com. TEICOPLANIN 100 MG POWDER AND SOLVENT FOR SOLUTION FOR INJECTION/ INFUSION OR ORAL SOLUTION. https://www.drugs.com/uk/teicoplanin-100-mg-powder-and-solvent-for-solution-for-injection-infusion-or-oral-solution-leaflet.html. Accessed 13 October 2020.
